# Flue Gas Desulfurization (FGD) Wastewater Treatment Using Polybenzimidazole (PBI) Hollow Fiber (HF) Membranes

**DOI:** 10.3390/membranes11060430

**Published:** 2021-06-05

**Authors:** Michael Dean Wales, Eminet Gebremichael, Xiao Wang, Elisabeth Perea, Palitha Jayaweera, Indira Jayaweera

**Affiliations:** SRI International, 333 Ravenswood Avenue, Menlo Park, CA 94025, USA; eminet.gebremichael@gmail.com (E.G.); xiao.wang.chem@gmail.com (X.W.); elisabeth.perea@sri.com (E.P.); palitha.jayaweera@sri.com (P.J.); indira.jayaweera@sri.com (I.J.)

**Keywords:** flue gas desulfurization (FGD), wastewater, polybenzimidazole (PBI), hollow fiber (HF), reverse osmosis (RO), coal fired power plant, desalination, membrane systems, Arrhenius equation, activation energy

## Abstract

Polybenzimidazole (PBI) hollow fiber membranes were used to treat flue gas desulfurization (FGD) wastewater (WW) from a coal fired power plant. Membranes were tested using both single salt solutions and real FGD WW. The PBI membranes showed >99% rejection for single salt solutions of NaCl, MgCl_2_, CaSO_4_, and CaCl_2_ at approximately 2000 PPM (parts per million). The membranes also showed >97% rejection for FGD WW concentrations ranging from 6900 to 14,400 PPM total dissolved solids (TDS). The pH of the FGD WW was adjusted between 3.97–8.20, and there was an optimal pH between 5.31 and 7.80 where %rejection reached a maximum of >99%. The membranes were able to operate stably up to 50 °C, nearly doubling the water flux as compared to room temperature, and while maintaining >98% salt rejection.

## 1. Introduction

In 1948, the US Federal Water Pollution Control Act was established to restore and maintain the health of the nation’s waters [1]. In 1972, this act was expanded and reorganized into what is commonly known as the “Clean Water Act” (CWA) [2]. Then in 1974, for the first time, the Environmental Protection Agency (EPA) issued Effluent Limitations Guidelines (ELGs) for the Steam Electric Power Generating Point Source Category [3]. The ELG standards were subsequently revised in 1977 and 1982, but not again until 2015 [3]. This gap of more than three decades was significant because between 1982 and 2015, new pollution controls were implemented at power plants and defined new sources of wastewater (WW) from flue gas mercury, selective catalytic reduction (SCR), and flue gas desulfurization (FGD). This study focuses on the treatment WW created from the FGD stream of coal fired power plants (CFPPs).

**Figure 1:**
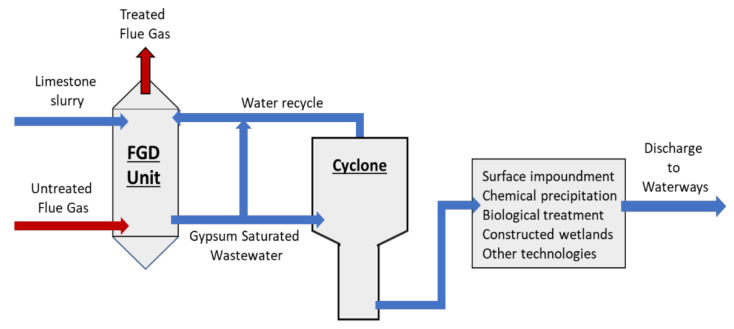
Simplified FGD WW treatment process. Gypsum-saturated WW exits the FGD unit and typically goes through a cyclone or settling process, followed by one or more treatment processes to remove pollutants. The treated FGD WW is eventually discharge to the environment.

The most common form of FGD is the wet scrubber [4,5,6,7]. The process consists of scrubbing the sulfur dioxide (SO_2_)-containing flue gas with a wet slurry of limestone (calcium carbonate, CaCO_3_). The SO_2_, CaCO_3_, and water react to produce a gypsum-saturated (CaSO_4_·2H_2_O) WW stream [5]. In addition to the gypsum, the FGD WW contains high amounts of chloride, nitrogen and nitrates, organics, various metals (some at trace levels, others in high concentrations), metalloids, and other nonmetals; with the exact composition depending on the origin of the coal [3,6,8]. Typical FGD WW treatments include a cyclone/hydrocyclone separation step followed by one or more treatment technologies: surface impoundments, chemical precipitation, biological treatments, evaporation systems, constructed wetlands, zero-discharge systems, and other technologies [3,8], Figure 1. In most cases, the treated FGD WW is eventually discharged into various waterways; therefore, the removal of pollutants is essential as many are detrimental to humans and/or ecological systems [9,10,11,12]. Unfortunately, standard treatment methods possess one or more deficiencies that make them either physically unable to meet the 2015 ELG standards or cost prohibitive. As such, the EPA has either rolled back or postponed implementation of several of the 2015 ELG standards relating to FGD WW [13,14]. Pressure driven membrane processes offer promising alternatives for FGD WW treatment with the potential to meet the initial 2015 standards.

Among the pressure driven membrane processes, reverse osmosis (RO) is a proven approach for treating high conductivity WWs and has many advantages, including the following:

- a modular design,
- high water permeability,
- the ability to process water of varying quality,
- continuous operation at room temperature,
- no need for energy-intensive phase changes.

As such, RO has been considered for a wide range of WW treatments and reclamation, from the steel industry to pharmaceutical and personal care products to radioactive wastewater [15,16,17,18,19,20,21]. At present, however, RO studies using FGD WW are limited [22]. Membranes for use in FGD WW must operate in a wide range of temperatures, osmotic pressures, pH values, and in the presence of organic, inorganic, and heavy metal pollutants; membranes that meet these demands are not currently available on the market. A polymer with the potential to meet these demands is polybenzimidazole (PBI).

PBI is heterocyclic polymer comprised of benzimidazole units, it is known for its high chemical and thermal resistance and can be fabricated into membranes [23,24]. Because of this, PBI membranes have been used in a wide range of applications, including those for fuel cells [25], high-temperature gas separations [26,27,28], nano filtration [29], and forward osmosis [30]. Our group has previously developed PBI hollow fiber membranes for use in gas separation and desalination applications [31,32,33,34,35]. However, to the best of our knowledge, this is the first time that PBI has been used for treating FGD WW.

The purpose of this study was to test the ability of PBI HF to treat FGD WW. The PBI membranes were first used on single-component salts of NaCl, MgCl_2_, CaSO_4_, and CaCl_2_ at approximately 2000 PPM (parts per million) and then tested with increasingly concentrated FGD WW from 6900 PPM TDS (total dissolved solids) up to 14,400 PPM. The effects of temperature, pressure, pH, and TDS on water flux and %rejection are reported.

## 2. Experimental

### 2.1. PBI Membrane and Modules

**Figure 2:**
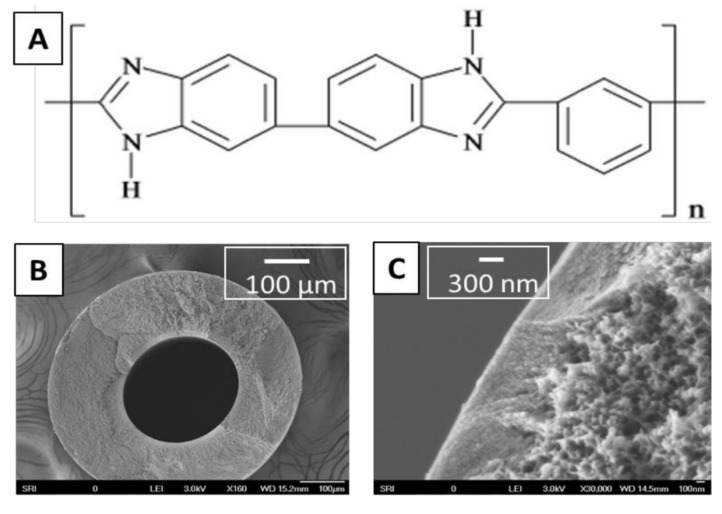
PBI hollow fibers. (**A**) The PBI monomer. (**B**) The SEM cross section of a single fiber. (**C**) The SEM image of the cross section near the membrane skin.

The PBI dope solution was obtained from PBI Performance Products Inc., and the chemical structure is shown in Figure 2. The dope solution consists of PBI, polyvinylpyrrolidine (PVP), dimethylacetamide (DMAc), and isopropyl alcohol (IPA). The bore solution and coagulation bath are non-solvent mixtures of IPA and methanol. The membrane formed has an integrally skinned asymmetric structure, with the dense skin layer on the shell (outer) side and the porous support on the lumen side (inside); the full fabrication method is published elsewhere [32]. The produced membranes have a 275-μm inner diameter (ID) and 550-μm outer diameter (OD). The membrane modules were made by potting 100 fibers, 30-cm long, for a total surface area of 0.052 m^2^. 

### 2.2. Testing System and Analysis

**Figure 3:**
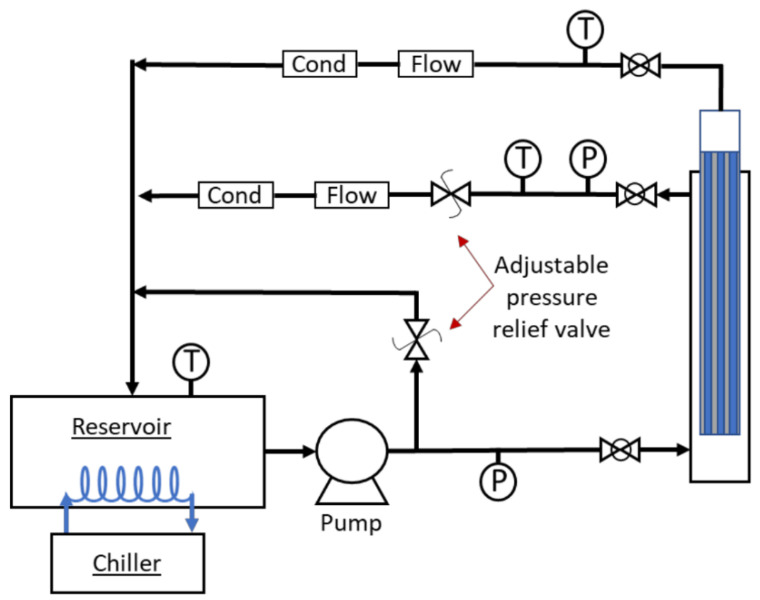
Simplified schematic of the RO test system: P = pressure transducer, T = K-type thermal couple, Flow = flow meter, and Cond = conductivity meter.

The RO testing system consists of a reservoir tank, a high-pressure pump (Hydra-Cell, model D-10), flow meters (Blue-White Industries, model F-400), conductivity meter (Amber Science, model 858 flow cell), pressure transducer (OMEGADYNE, model PX319-aKG5V), K-Type thermocouple (Omega), and the RO module. Transmembrane pressure was achieved using a back-pressure regulator actuated by a pressurized N_2_ tank, Figure 3. A chiller (NESLAB, model RTE-110) was used to keep the solution at a constant temperature. Both the permeate and retentate were recycled back to the solution reservoir, keeping the test solution at a constant concentration. Water flux through the membrane was determined gravimetrically, a graduated cylinder was placed on top of a balance, and the mass of permeate collected (M) was measured over a recorded time interval (t); the surface area (A) was determined as previously described. The permeate flux is reported as follows:

(1) $$Flux\left(\frac{L}{m^{2}*hr}\right)=\frac{V}{A*t}$$

where *m^2^* is meters squared and *hr* is hour (LMH). Prior to initial use, membranes were soaked in deionized water overnight. In between experiments, the membranes were cleaned by rinsing with deionized (DI) water.

**Figure 4:**
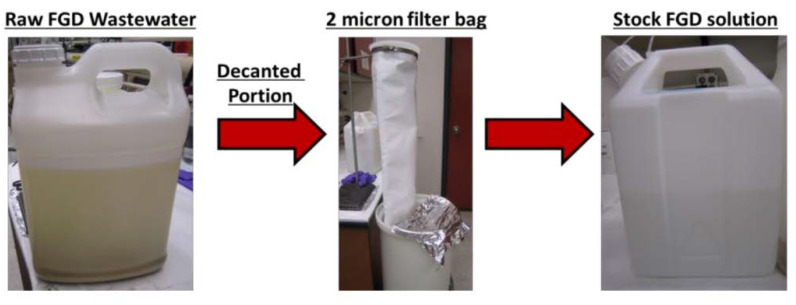
Preparation of stock FGD WW solution. Raw FGD WW was allowed to settle and then decanted. The decanted portion was filtered through a filter bag to produce the stock FGD solution. This stock solution was diluted for RO studies.

Raw FGD WW was obtained from a coal-fired power plant (CFPP) with a wet FGD system operating in Illinois, Table 1. Prior to testing, the WW was allowed to settle and then decanted. The decanted portion of the solution was then filtered through a 2-micron polypropylene microfiber filter bag (POMF-2A-P2P), Figure 4. The decanted and filtered FGD WW is referred to as the FGD stock solution; it was diluted and used for RO studies. In addition to the FGD WW, solutions of sodium chloride (NaCl), magnesium chloride (MgCl_2_), calcium sulfate (CaSO_4_), and calcium chloride (CaCl_2_) were made by dissolution of the appropriate salt in DI water. All concentrations were determined using a Cole-Parmer conductivity meter (catalog# EW-19601-03). The %rejection was determined as follows:

(2) $$\%Rejection=\left[1-\frac{C_{p}}{C_{f}}\right]*100,$$

where *C_p_* is the permeate concentration, *C_f_* is the feed concentration. The conductivity meter was calibrated for both single salts (NaCl, MgCl_2_, CaSO_4_, and CaCl_2_) and FGD WW using 5-point calibration curves, all calibrations have an R^2^ > 0.99.

Scanning electron microscope (SEM) images were obtained using a field emission scanning microscopy, JEOL-6700, in lower secondary electron (LEI) mode, an accelerating voltage of 3 KV, and a probe current of 20 µA.

## 3. Results and Discussion

### 3.1. Single Salt Experiments (Module 1)

Before we tested the FGD WW, we used Module 1 for single-component rejection and flux measurements for NaCl, MgCl_2_, CaSO_4_, and CaCl_2_. Each salt solution was made by dissolving a known amount of each salt in DI water and stirring with a magnetic stir bar overnight. The system was allowed to equilibrate for at least 60 min before we recorded the measurements. We screened the membranes for water flux and %rejection at a pressure of 367 ± 5 psig and temperature of 20 ± 1 °C. The initial results showed >99% rejection for all salts and water flux ranging from 1.2–1.5 LMH, Table 2.

**Figure 5:**
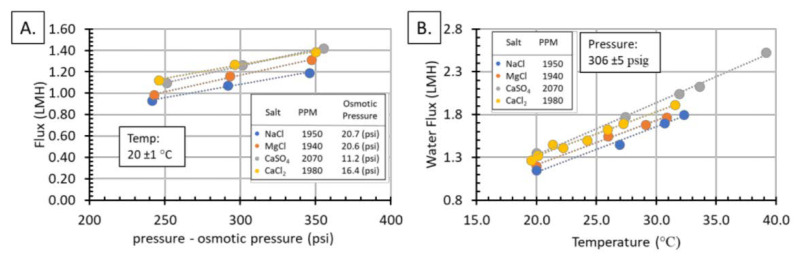
Water flux increased linearly with pressure (**A**) and temperature (**B**). This is expected behavior for RO membranes. The order of water flux by salt solution was CaSO_4_ > CaCl_2_ > MgCl_2_ > NaCl.

Additionally, the permeate flux and %rejection of the salts were measured as a function of temperature and pressure. Figure 5 shows permeate flux increases linearly with both temperature and pressure in the range tested, which is expected for polymeric RO membranes [36]. Water flux and pressure differences are directly proportional according to Equation (3):

(3) $$J_{w}=A\left[\Delta P-\Delta \pi \right],$$

where *J_w_* is the water flux, *A* is a constant, Δ*P* is the pressure difference, and Δ*π* is the osmotic pressure difference. However, temperature effect on water flux is an activated process that is governed by an Arrhenius relationship, which can have more dramatic effects than pressure on the water flux. Figure 5B shows increasing from 20 to 40 °C doubles the water flux. The order of water flux according to salt solution was CaSO_4_ > CaCl_2_ > MgCl_2_ > NaCl. This flux order can be explained because it is the reverse order of the osmotic pressure, where the Δπ of CaSO_4_ < CaCl_2_ < MgCl_2_ < NaCl, Figure 5. The membrane module showed a %rejection of >99% for all data shown in Figure 5.

### 3.2. FGD WW–Temperature and Pressure Effects (Module 1)

Following the single salt solutions, the same membrane module (Module 1) was exposed to increasingly concentrated FGD WW. The FGD WW was minimally treated, only decanted and passed through a filter bag (process detailed previously), and diluted to the reported concentrations: 6900, 8100, 11,000, and 14,000 TDS, reported as PPM. Similar to the single salt solutions, the pressure and temperature were varied and the flux and %rejection were measured.

**Figure 6:**
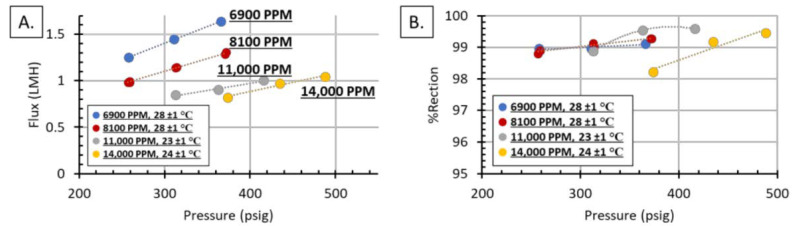
(**A**) Flux as a function of pressure for diluted FGD wastewater. Flux increased linearly with pressure, and flux decreased with increasing concentration. (**B**) %Rejection as a function of pressure.

As expected, water flux and %rejection increased with increasing pressure, Figure 6. Although both water and solutes have increased fluxes with increasing pressure, the water flux increases at a faster rate than the solutes, causing an increase in %rejection. Other expected trends include decreasing water flux with increasing solute concentration and decreasing %rejection with increasing concentration. This is because increasing the salt concentration increases the osmotic pressure, which decreases the water flux. The %rejection decreases because salt flux is governed by Equation (4):

(4) $$J_{s}=B\left[\Delta C\right],$$

where *J_s_* is the salt flux, *B* is the permeability constant, and *ΔC* is the salt concentration difference across the membrane [36]. As the concentration of the FGD WW increases, the ΔC and salt flux increases, which decreases the %rejection; additionally, with fixed hydraulic pressure, elevation of salt concentration leads to decline of water flux, which also contributes to the decay of salt rejection. Condid et al. found similar results testing two commercially available thin-film composite polyamide membranes [22].

**Figure 7:**
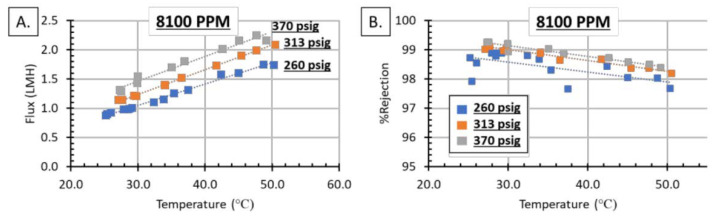
The FGD WW at 8110 PPM: (**A**) Flux increased linearly with temperature in the pressure and temperature range tested. Additionally, flux increased with increasing pressure. (**B**) %rejection decreased with increasing temperature and increased with increasing pressure.

**Figure 8:**
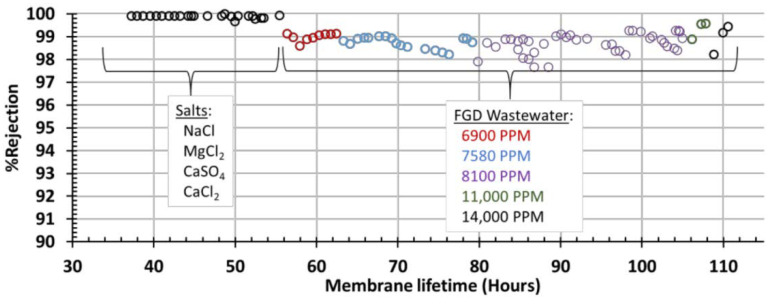
The image shows the %rejection for all reported runs on Module 1. The membrane showed near complete rejection for the single-component salt solutions and >97% under all operating conditions for the real FGD WW.

Figure 7 shows temperature and pressure effects on 8100 PPM FGD WW. Similar to the single-salt solutions, increasing temperature increases water flux; however, the real FGD WW sees a decrease in %rejection with increasing temperature. This is likely due to the elevated TDS levels, and, again, is expected behavior for polymeric RO membranes. This initial screening of the membranes was very successful: all of the single salt solutions showed >99% rejection, and a majority of the FDG WW tests had >98% rejection. All four FGD concentrations tested were able to be filtered at >99% rejection under specific temperature and pressure ranges. Furthermore, this module (Module 1) was operated for over 100 h, over a variety of testing conditions without leaking, Figure 8. Figure 8 shows the chronological order of experiments represented in Figure 5, Figure 6 and Figure 7, and does not represent a single, continuous long-term study.

### 3.3. FGD WW–pH Effects (Module 2)

Following the successful screen of Module 1, we tested the effect of pH on a second module from the same batch of membranes (Module 2). pH is an important parameter, as it can affect the surface charge of the membrane [29,37,38] and the chemistry of the FGD WW [6]. The FGD WW composition is not universal: different coal sources and different power plants produce FGD WW with different compositions and pH values. The RO membranes need be flexible enough to handle a wide range of FGD WW. The decanted and filtered FGD WW started at a pH value of 6.80 and TDS of 14,400 PPM. We adjusted the solution by adding concentrated HCl or NaOH. The pH adjustments raised the TDS concentration slightly but not significantly; the highest TDS was 15,100 PPM or <5% increase.

**Figure 9:**
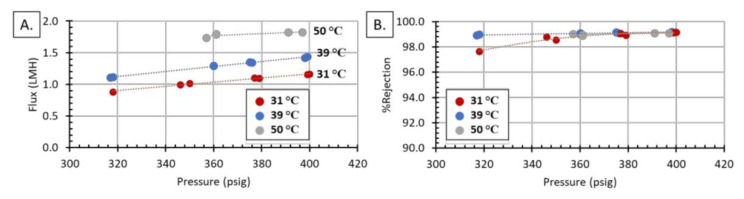
Module 2 characterization using 14,400 PPM FGD wastewater. Effects of temperature and pressure on: (**A**) water flux and (**B**) %rejection.

Before adjusting the pH, the membrane was screened with the stock FGD solution by changing temperature and pressure, Figure 9. Module 2 was subject to the same trends as Module 1; there was increasing flux with increasing temperature and pressure, and increasing %rejection with increasing pressure. Module 2 also demonstrated the ability to reach >99% rejection at temperatures up to 50 °C; this is noteworthy because operating at elevated temperatures increases the water flux, which increases the filtering rate. The effect of temperature on membrane processes has been thoroughly reported on in the literature [39,40,41,42,43]. The temperature can affect concentration polarization, salt rejection, feed pressure, feed flow, and water recovery; however, most importantly, feed temperature is known to be directly proportional to water flux. The ability to operate at higher temperatures is also important from a cost perspective. Both Sassie et al. [40] and Nisan et al. [43] have developed models that demonstrate that increasing the operating temperature of the feed water can decrease the cost of running a desalination plant. Sassie et al. demonstrated that by increasing the feed temperature they were able to decrease the feed pressure, reduce electrical usage, and reduce the number of membrane stages [40]. Operating at reduced pressures and with a reduced number of stages lowers both the operating cost and the initial capital investments, as fewer modules and pumps would be required.

**Figure 10:**
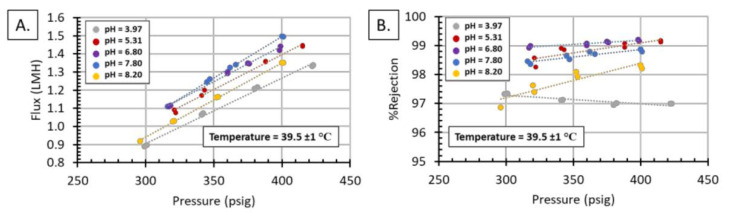
Effects of pressure and pH on: (**A**) water flux and (**B**) %rejection for FGD wastewater.

**Figure 11:**
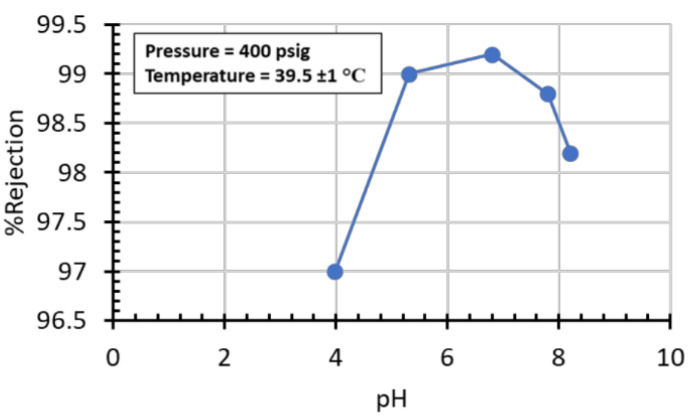
The %rejection reached a maximum between pH 5.31 and 7.8.

Following this screening, we tested the FDG WW at different pH values. The water flux followed the expected trend of increasing water flux with increased pressure; however, pH did have a noticeable effect on the flux rate, Figure 10. The highest flux was found at a pH of 7.80 and the lowest at a pH of 8.20, with a 16% difference in flux at 400 psig. The %rejection did not follow a specific trend, plotting %rejection as a function of pH showed there is an optimal pH at which the %rejection equals a maximum, Figure 11. Similar results can be found throughout the literature, and the behavior has been attributed to the composition of the WW feed and not to the surface charge of the membrane [44,45,46,47]. Qin studied the effect of feed pH on three different RO membranes, with different isoelectric points, and found the pH of maximum %rejection was the same for all membranes [45], indicating the pH of maximum %rejection is independent of membrane isoelectric point, and instead is a function of the feedwater composition. Hyung found the same results for boron rejection of seawater desalination using six different commercial membranes [46]. This idea is further supported by research by Park et al.; this team determined that membrane surface charge is not the dominant factor in individual ion rejection for RO processes [47].

**Figure 12:**
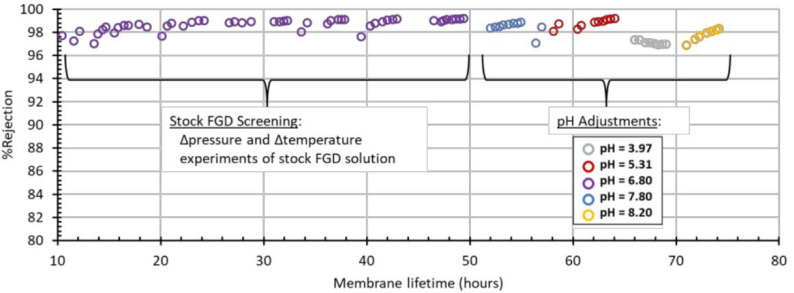
The image shows the %rejection for FGD wastewater of varying pH on Module 2. The membrane showed >96% rejection for all pH values and operating conditions tested. Furthermore, the membrane was able to reach >98% rejection for all pH values under specific conditions.

Figure 12 shows the stability of Module 2 over the lifetime of the membrane. The PBI RO membranes were able to operate in real FGD WW over a range of pH values for 75+ h. The membrane showed >96% rejection for all pH values and operating conditions tested. Furthermore, the membrane was able to reach >98% rejection for all pH values under specific conditions. Figure 12 shows the chronological order of experiments represented in Figure 9, Figure 10 and Figure 11, and does not represent a single, continuous long-term study. We planned on conducting a long-term study of this membrane to determine flux and %rejection over time, while holding temperature, pressure, and pH constant; unfortunately, the membrane was damaged during handling and we were unable to carry out that study.

### 3.4. Activation Energy

The data from Figure 5B was used to calculate the single salt apparent activation energies (E_a_), Table 3. The activation energies were determined by plotting the ln(permeability) vs. 1/T; where permeability is pressure-normalized flux ($\frac{liter}{m^{2}hrpsi}$) and T is temperature in kelvin. The resulting Arrhenius plot have a slope equal to -E_a_/R; where R is the molar gas constant. The order of activation energy for the single salts are CaSO_4_ < CaCl_2_ < MgCl_2_ < NaCl, which is the reverse order of osmotic pressure (the same order as water flux). This is because as temperature is increased, the osmotic pressure also increases. As Equation (3) shows, the net pressure different (NPD) across the membrane is the hydraulic pressure difference (ΔP) minus the osmotic pressure (Δπ). For a constant pressure, but increasing temperature, the osmotic pressure increases, which lowers the NPD. This physically shows up as a lower permeability for equal ΔP, which explains the E_a_ order of the single salts. Table 3 also shows the E_a_ for 8100 ppm FGD WW shown in Figure 7. The FGD WW shows that increasing pressure decreases the Ea; this means that permeability is increasing with increased pressure. This pattern would indicate that concentration polarization is likely not an issue. Concentration polarization would cause an increase in osmotic pressure, which would decrease the permeability of the solution. The increased permeability in both the single salt and 8100 ppm FDG WW are most likely due to a decrease in viscosity, which increases water permeability [41,42,48]. It should be noted that activation energy depends on multiple variables, including viscosity, pH, solutes present in solution as well as solutes permeating through. Furthermore, the overall activation energy is made up of many components, such as non-viscous contributions (as an example). However, the parsing of the individual components to the activation energy are beyond the scope of this study.

## 4. Conclusions

This study successfully demonstrated the stable operation of a single-stage RO system for treating concentrated FGD WW with 97% salt rejection. The PBI HF membranes were first tested with single salt solutions of CaSO_4_, CaCl_2_, MgCl_2_, and NaCl at approximately 2000 PPM each. These single-component salt solutions all showed >99% rejection at operating conditions up to 365 psig and 40 °C. Following testing of the single-component salts, the membranes were tested using minimally pretreated FGD WW from a coal-fired power plant using increasingly concentrated solutions with TDS values of 6900 to 15,000 PPM. The water flux was found to increase linearly with pressure and temperature and the %rejection also increased with increasing pressure and temperature. It is notable that our system was able to operate stably at 50 °C, resulting in a near doubling of water flux compared to room temperature. Commercial membranes operate at room temperature, while the PBI membrane is extremely thermally stable and has a high glass-transition temperature that allows operation at higher temperatures and fluxes. The effect of feed pH was also tested, and there was an optimal pH at 6.80 where %rejection reached a maximum of >99%. The results from this study indicate that using PBI hollow fiber membranes in a multistage system can allow a high water recovery for FGD WW treatment. Future work includes the effect of flow rates on concentration polarization, fouling, scaling, and how these factors affect each other.

## Figures and Tables

**Table 1:** The FGD WW Characterization.

Property	Type/Value
FGD type	Wet FGD
Coal type	Subbituminous
Total TDS	≈15,000 PPM
Total organic carbon	81 PPM
Chloride/sulfate ratio (ppm/ppm)	≥5

**Table 2:** Initial Membrane Screening–Water Flux and Salt Rejection.

Salt	Concentration (ppm)	Water Flux (LMH)	Osmotic Pressure (psi)	Rejection
NaCl	1950	1.19	21.4	>99%
MgCl_2_	1940	1.31	20.6	>99%
CaCl_2_	1980	1.39	16.4	>99%
CaSO_4_	2070	1.42	11.2	>99%

Pressure = 367 ± 5 psig. Temperature = 20 ± 1 °C.

**Table 3:** Apparent Activation Energy for Water Permeability.

Solution	Pressure (psig)	Slope (–E_a_/R)	R^2^	E_a_ (kJ/mol)	Temperature Range (°C)
NaCl	306	−3588.0	0.980	29.8	20−30
MgCl_2_	306	−3451.6	0.996	28.7	20−30
CaCl_2_	306	−3111.6	0.982	25.9	20−30
CaSO_4_	306	−3067.1	0.998	25.5	20−40
81,000 ppm FGD WW	260	−2756.0	0.993	22.9	25−50
81,000 ppm FGD WW	315	−2554.3	0.992	21.2	25−50
81,000 ppm FGD WW	373	−2403.4	0.957	20.0	25−50

## Data Availability

Not applicable.
